# Primary health care approach to Diabetes mellitus in Malawi

**DOI:** 10.11604/pamj.2014.18.261.2948

**Published:** 2014-07-29

**Authors:** Abbas Abdalrahman Assayed, Imied Daitoni

**Affiliations:** 1 Mama Khadijah Medical Centre, Namwera, Mangochi, Malawi; 2 Health management unit, Department of Community Health, College of Medicine, University of Malawi, Blantyre, Malawi

**Keywords:** Diabetes, primary health care, Malawi

## Opinion

Globally, Dm is becoming a major public health problem and mostly affecting the low and middle-income countries. It is estimated that 285 million people live with diabetes worldwide and expected to hit as high as 436 million by 2030 [1]. In Africa about 12 million were estimated to have Dm in the year 2010 and this number has increased to 14.7 million in 2011 [2]

The WHO STEP wise approach to chronic disease risk factor surveillance estimated the prevalence of Dm in Malawi to be 5.6% among the age group 25-64 in the year 2009 [3]. A prevalence of 5.6% is an alarming level which requires the government of Malawi, through the ministry of health to innovatively respond to this new epidemic. To better handling this health problem, it is wise to adopt feasible and pragmatic approach putting in mind the position of Malawi on the world economic and health classification. In this view point, we strongly recommend this approach so as to mitigate the morbidity and mortality of diabetes in Malaawi.

### The Malawi Government position On Diabetes

Until the year 2011 there was no specific directorate in the Ministry of Health to address the Non-communicable Diseases (NCD), but this year the directorate has been established and the NCD (including Dm) have been prioritized in the Essential health Package (EHP) [4] in the Health Strategic Plan (HSSP) 2011-2016, and are tabled among the National Research Agenda.

In Malawi there are 28 districts with a hospital in each. There also five zones with a referral hospital in each zone. Diabetes services are provided in almost all district hospitals. It is unknown whether the district hospitals will continue to offer such services efficiently in the near future as information about sustainability or quality of care is not there (at the time of the development of this paper). So, Malawi health authorities are yet to have a well established an effective care services and sound health polices with reasonable human resource and materials. The ideal Dm care requires a wide range of resources and protocols starting from health education and life-style and behavioural change to a robust early detection and diagnosis, timely and effective management as well as prevention and treatment of potential inevitable complications. So in this paper we will explain how the simplistic Primary Health Care (PHC) Approach could be a better and feasible way out.

### Primary health care features and capability to address diabetes

Though the PHC is the currently functioning system, there are some challenges facing the implementation such as accessibility to health services and shortage of resources. [5] The PHC was adopted during the WHO meeting in 1978 as key approach to the health of the world. PCH emphasizes and advocates for the points that formulate the concept. These can be summarized as; access, equity, essentiality, appropriate technology, multisectral collaboration, and community participation and empowerment. As thus, the concept comprehensively clears some worries that might be raised in regard to the human resources and materialistic resources and even the technical capacity.

### Successful stories used low-cost interventions in rural settings

Diabetes care in resource limit settings can be optimised with more innovative ways with no much costs implications. A nurse-led service, for instance, has improved retention of patients in remote areas. There are several studies showed that a Dm single recall system managed by local healthcare workers and supported by a diabetes out-reach service has good results in improving care and reducing hospitalization in a high risk population. This teaches us that diabetes could be handled in rural areas. In areas where people are underserved this new approach could bring good results, especially in areas like rural Malawi. This approach will also facilitate the integration of care for some Non-Communicable Diseases (NCDs) such as Hypertension, Asthma and Epilepsy at low costs and non-physician clinicians and nurses.

### Health education and diabetes

Health education for patients with type 2 diabetes is an efficient tool in improving care without affecting the health care costs. Health care costs cause real qualms in most Africa, so with this approach financial allocations will be so minimal. Health education, amazingly, has wonderful positive results in most of the diabetes care angles. It helps in improving the self-care and life-style.

It has been appreciated that diabetes care in the community is a vital component in the PHC. Community diabetes care relies mostly on health information, education and communication which are very effective and at the same time it is cost-effective. Using health education as a PHC tool is found to be a potential risk reducer and it helps preventing and delaying onset of the major diabetes complications.

Human Resource and Training for Diabetes CareThe main drive for diabetes care is not only the availability and adequacy of the healthcare work force but the level of knowledge and skills required for caring patients. This because not all healthcare workers know about diabetes care, rather it requires special trainings and mentoring by experienced health providers. Data on the knowledge about diabetes among the healthcare workers in Malawi is not available, however, literature from other parts of Africa show that most of the Healthcare workers lack knowledge and skills on diabetes care provision. Hence it is not surprising that primary care for diabetes and hypertension in the public health centres is suboptimal and in Malawi the glycemic and complications control is poor even in zonal referral hospital settings. It is of prime importance that policy makers and planners need to provide training and refresher courses on diabetes care according to the most recent guidelines.

The current service delivery system in Malawi has no capacity to address the Dm burden in the rural areas as the accessibility and availability of the healthcare generally is a real challenge. However, we have tried to explain how Malawi could do much more to tackle diabetes and its complications if the PHC approach is adequately employed and sustained.
